# High-Lard and High-Fish-Oil Diets Differ in Their Effects on Function and Dynamic Behaviour of Rat Hepatic Mitochondria

**DOI:** 10.1371/journal.pone.0092753

**Published:** 2014-03-24

**Authors:** Lillà Lionetti, Maria Pina Mollica, Immacolata Donizzetti, Giorgio Gifuni, Raffaella Sica, Angelica Pignalosa, Gina Cavaliere, Marcello Gaita, Chiara De Filippo, Antonio Zorzano, Rosalba Putti

**Affiliations:** 1 Department of Biology, University of Naples “Federico II”, Naples, Italy; 2 Departament de Bioquímica i Biologia Molecular, Facultat de Biologia, Universitat de Barcelona, Barcelona, Spain; University of Mississippi, United States of America

## Abstract

**Background:**

Mitochondria are dynamic organelles that frequently undergo fission and fusion processes, and imbalances in these processes may be involved in obesity and insulin resistance.

**Aims:**

The present work had the following aims: (a) to evaluate whether the mitochondrial dysfunction present in the hepatic steatosis induced by a high-fat diet is associated with changes in mitochondrial dynamics and morphology; (b) to evaluate whether effects on the above parameters differ between high-lard and high-fish-oil diets, as it has been suggested that fish oil may have anti-obesity and anti-steatotic effects by stimulating fatty acids utilisation.

**Methods:**

The development of hepatic steatosis and insulin resistance was monitored in rats fed a high-lard or high-fish-oil diet. Immunohistochemical and electronic microscopic observations were performed on liver sections. In isolated liver mitochondria, assessments of fatty acids oxidation rate, proton conductance and oxidative stress (by measuring H_2_O_2_ release and aconitase activity) were performed. Western blot and immunohistochemical analyses were performed to evaluate the presence of proteins involved in mitochondrial dynamics (i.e., fusion and fission processes). To investigate the fusion process, mitofusin 2 and autosomal dominant optic atrophy-1 (OPA1) were analysed. To investigate the fission process, the presence of dynamin-related protein 1 (Drp1) and fission 1 protein (Fis1) was assessed.

**Results:**

High-lard feeding elicited greater hepatic lipid accumulation, insulin resistance with associated mitochondrial dysfunction, greater oxidative stress and a shift towards mitochondrial fission processes (versus high-fish-oil feeding, which had an anti-steatotic effect associated with increased mitochondrial fusion processes).

**Conclusions:**

Different types of high-fat diets differ in their effect on mitochondrial function and dynamic behaviour, leading to different cellular adaptations to over-feeding.

## Introduction

Mitochondrial dysfunction is characteristic of both insulin resistance (IR) and non-alcoholic fatty liver disease (NAFLD) [1], [2]. In conditions involving IR, impaired lipid oxidation in the liver has been reported [2], and in NAFLD, an elevated lipid flux stimulates hepatic fat oxidation, which is associated with electron chain impairment and results in excessive formation of reactive oxygen species (ROS) [3]–[5]. Mitochondrial respiratory chain impairment may be associated with ultrastructural damage, as found in individuals with non-alcoholic steatohepatitis (NASH) [6]. Indeed, the functions of mitochondria correlate well with their structure and morphology. Mitochondria may exhibit a tubular or fragmented morphotype, or they may be assembled into networks, with their distribution and morphology frequently altered by recurrent fission and fusion events in response to both cellular energy demands and environmental challenges [7]–[9]. Several proteins regulate these dynamic processes. Fusion is coordinated by mitofusin 1 and 2 (Mfn1 and Mfn2, respectively) and by autosomal dominant optic atrophy-1 (OPA1) [9]–[11], and these proteins are associated with the mitochondrial outer and inner membranes, respectively. With regard to fission, dynamin-related protein 1 (Drp1) is recruited to mitochondria to mediate fission activities, and fission 1 protein (Fis1), an integral outer mitochondrial membrane protein, plays an essential role in completing the fission process [8]–[9]. Maintaining normal mitochondrial morphology by tilting the balance between fusion and fission may be important in the regulation of mitochondrial energetics [7]–[9]. Moreover, a reduction in mitochondrial fusion may be involved in the development of obesity and IR [12]. Furthermore, fission inhibition and/or fusion activation have been found to counteract many of the disease phenotypes related to IR and diabetes [13], whereas enhanced fission machinery has been found in skeletal muscle in genetically obese and diet-induced obese mice [14] as well as in the db/db mouse liver alongside decreased mitochondrial respiratory capacity [15].

The present study aimed to determine whether mitochondrial dysfunction in the rat liver induced by a high-fat diet is associated with changes in the mitochondrial fusion/fission balance. In particular, we compared the effect of different dietary fat sources on the above parameters, as fish oil feeding (in contrast to saturated fatty acids feeding) has anti-obesity and anti-steatotic effects [16], [17] as well as potential benefits for IR [18]. In rodents, fish oil feeding prevents lipid accumulation in white adipose tissue (WAT) [16], [19] by limiting the triglyceride supply to WAT through increased PPAR alpha-mediated fatty acids oxidation in the liver. In fact, it has been suggested that PPARα activation in the liver might be related to the anti-obesity and anti-steatotic effects of fish oil feeding [16].

The aim of the present study was to compare the effects on hepatic mitochondrial function and dynamic behaviour between a high-lard (HL) diet (mainly saturated fatty acids) and a high-fish-oil (HFO) diet (mainly omega-3 polyunsaturated fatty acids; PUFAs). Our results suggested that these two high fat diets elicited different degrees of hepatic steatosis in treated rats with different behaviours in the mitochondrial dynamics and bioenergetics, indicating that a HFO diet may lead to a lower degree of hepatic steatosis through mitochondrial fusion and the amelioration of mitochondrial fatty acids utilisation.

## Materials and Methods

### Ethics statement

This study was performed in strict accordance with the criteria established by the National Institutes of Health. The protocol was approved by the Committee on the Ethics of Animal Experiments of the University of Naples “Federico II” (Permit Number: 2010/0149862). All surgeries were performed under chloral hydrate anaesthesia, and all efforts were made to minimise suffering.

### Materials

The analytical-grade chemicals used were purchased from Sigma (St. Louis, MO, USA). The fish oil used in this study was cod liver oil (New.Fa.Dem. srl, Giugliano, Naples, Italy).

### Animals and diets

Male Wistar rats aged 60 days (Charles River Italia, Calco, Como, Italy) were caged singly in a temperature-controlled room at 24°C with a 12 h light–dark cycle. The rats were divided into 3 groups (8 rats each), and in each group, the mean body weight was approximately 400 g. For 6 weeks, the groups were fed the following diets: the first group (N) received a standard diet (10.6% fat J/J); the second group (L) received the HL diet (40% fat J/J); and the third group (F) received the HFO diet (40% fat J/J). Diet compositions are shown in Table 1. The two high-fat diets were formulated to differ from the standard low-fat diet with regard to the contributions of fat and carbohydrate to the energy value but to be identical in terms of proteins, vitamins, minerals and fibres. The body weights and food intake were monitored daily to enable calculations of gain in body weight and energy intake. The energy contents of the standard, HL and HFO diets were determined by bomb calorimetry.

**Table 1:** Composition of diets.

Component		Control diet	High-fat diet	
			High Lard (HL)	High Fish Oil (HFO)
			g/100 g diet	g/100 g diet
Standard feed g		100	51.03	51.03
Casein^a^ g		-	9.25	9.25
Lard g		-	21.8	-
Fish oil^b^ g		-	-	21.8
Sunflower oil g		-	1.24	1.24
AIN 76Mineral mix^c^ g		-	1.46	1.46
AIN 76Vitamin mix^d^ g		-	0.42	0.42
Choline bitartrate g		-	0.08	0.08
Methionine g		-	0.12	0.12
Energy density, kJ/g diet		15.88	20.00	20.00
Energy (J/100 J)				
	Protein %	29	29	29
	Lipid %	10.6	40	40
	Carbohydrate %	60.4	31	31

a Purified high-nitrogen casein containing 88% protein.

b Fish oil  =  Cod liver Oil.

c American Institute of Nutrition (1977).

d American Institute of Nutrition (1980).

The experimental design was repeated three times (using different rats each time) for all the required measurements to be made. In particular, groups of rats were fasted overnight to determine basal glucose and serum insulin levels. For the measurements of insulin-dependent AKT phosphorylation, rats that had fasted for five h were euthanised 15 min after an i.p. injection of insulin (homologue rapid-acting, 10 units/kg body wt; Novartis, Basel, Switzerland).

At the end of the dietary treatment, the rats were anaesthetised by injection of the abovementioned chloral hydrate (40 mg/100 g body weight, i.p.), and blood was taken via the inferior vena cava. The livers were removed, weighed and either immediately processed for isolation, processed for the morphological analysis of mitochondria or frozen in liquid nitrogen and stored at −80°C for later processing.

### Determination of serum parameters

Serum levels of triglycerides (TGs) and alanine aminotransferase (ALT) were determined using standard procedures [4]. Serum glucose and insulin values were determined by means of a glucose monitor (BRIO, Ascensia, NY) calibrated for use with rats and with ELISA (Mercodia rat insulin; Mercodia, Uppsala, Sweden), respectively.

### Hepatic lipid content

Lipid content was determined gravimetrically after extraction in chloroform–methanol and evaporation to constant weight with a rotating evaporator (Heidolh, Germany) according to the method described by Folch [20].

### Light microscopy and immunohistochemistry

Small pieces of liver were fixed in Bouin's fluid, embedded in Paraplast and cut into 4–5 μm sections. Immunohistochemical reactions were performed by the polymer Envision dual-link technique as previously described [21]. After antigen retrieval and quenching of endogenous peroxidase, sections were incubated overnight at 4°C with the following primary antisera (diluted 1∶300–1∶400): Mfn2, peroxisome proliferator-activated receptor-α (PPARα, Abcam, Cambridge, UK), Drp1 (Santa Cruz, CA, USA) and OPA1 (Novus Biologicals, Cambridge, UK). The sections were then incubated in Envision at RT, and immunostaining was performed using 3,3′-diaminobenzidine (DAB) as the chromogen. To test the specificity of the reagents, the following controls were performed: (a) omission of the primary antiserum and incubation of the sections with either non-immune serum (1∶10 and 1∶20) or bovine serum albumin (BSA; Sigma); (b) absorption of the optimally diluted primary antiserum with its specific peptide (10 nmol/ml of optimally diluted antiserum) for 24 h at 4°C. When specific peptides were used, the staining was abolished.

Images of sections were acquired using a Zeiss Axioskop microscope fitted with a TV camera.

### Electron microscopy (EM)

A conventional EM technique was used as previously described [22]. Briefly, small liver slices were fixed for 2 h at 4°C in 1% glutaraldehyde in Millonig buffer (pH 7.3). The slices were post-fixed in 1% osmium tetroxide, embedded in Epon 812 and cut using an ultra-microtome. Ultra-thin sections were mounted on copper grids and contrasted with uranyl acetate and lead citrate. In addition to ultra-thin sections, sections of 1–3 μm (semi-thin sections) were collected on glass slides and stained with toluidine blue. All the sections were examined using a CM 12 Philips electron microscope at the interdepartmental centre for electron microscopy (CISME).

### Morphometric analysis

For each liver, seventeen adjacent sections were acquired at 20,000× magnification. For each micrograph, we selected three different non-superimposed areas that were analysed for morphometric analysis, with care taken to ensure that areas of the analysed livers were the same size. The diameter of each mitochondrion (the long diameter for tubular mitochondria) was drawn interactively using Zeiss Axiovision image analysis software. The numbers of tubular, round and total mitochondria were determined. Data are expressed as the mean ± SE.

### Preparation of isolated mitochondria

Mitochondria were isolated from the liver as previously described [23]. Briefly, tissue fragments were gently homogenised with a medium (pH 7.4) containing 220 mM mannitol, 70 mM sucrose, 20 mM HEPES, 1 mM EDTA and 0.1% (w/v) fatty-acid-free BSA in a Potter Elvehjem homogeniser set at 500 rpm (4 strokes/min). The homogenate was then centrifuged at 1000 g_av_ for 10 min, and the resulting supernatant was again centrifuged at 3000 g_av_ for 10 min. The mitochondrial pellet was washed twice and finally resuspended in a medium (pH 7.0) containing 80 mM LiCl, 50 mM HEPES, 5 mM Tris-PO_4_, 1 mM EGTA and 0.1% (w/v) fatty-acid-free BSA. Enzymatic and electron microscopy characterisation has shown that this isolation procedure (centrifugation at 3000 g_av_ for 10 min) results in a cellular fraction, which is constituted essentially by mitochondria [24]. The protein content of the mitochondrial suspension was determined by the method of Hartree [25] using BSA as the protein standard.

### Mitochondrial parameters

Mitochondrial oxygen consumption was measured polarographically using a Clark-type electrode (Yellow Springs Instruments, Yellow Springs, Ohio) at a temperature of 30°C. Isolated mitochondria were incubated in a medium (pH 7.0) containing 80 mM KCl, 50 mM HEPES, 5 mM KH2PO4, 1 mM EGTA and 0.1% (w/v) fatty-acid-free BSA to oxidise their endogenous substrates for a few minutes. Substrates were then added at the following concentrations: 10 mM succinate plus 3.75 μM rotenone; 10 mM pyruvate plus 2.5 mM malate; or 40 mM palmitoyl-CoA plus 2 mM carnitine and 2.5 mM malate. State 4 oxygen consumption was obtained in the absence of ADP, and State 3 oxygen consumption was measured in the presence of 0.3 mM ADP. The respiratory control ratio (RCR) was calculated as the ratio between states 3 and 4 according to Estabrook [26]. Oxygen consumption rates (OCRs) in the presence of oligomycin (2 μg/ml) or FCCP (1 μM) were also measured to evaluate the ratio of the maximum OCR in the presence of FCCP to the OCR with oligomycin (OCR FCCP/OCR oligomycin).

The activity of the carnitine palmitoyltransferase (CPT) system (CPT1 plus CPT2) was measured spectrophotometrically at 412 nm [27].

### Measurement of basal proton leak

Mitochondrial oxygen consumption was measured polarographically with a Clark-type electrode, and membrane potential recordings were performed in parallel with safranin O using a JASCO dual-wavelength spectrophotometer [23], [28]. Safranin is a positively charged dye that undergoes spectral shifts upon its potential-dependent distribution between the external medium and the intramitochondrial compartment and on its staking to inner mitochondrial membrane anionic sites [29]–[30]. As previously described [23], [28], the quenching of the colour was measured at 511–533 nm, yielding an upward deflection for increased quenching. The absorbance readings were transferred to mV membrane potential using the Nernst equation as follows: Δψ = 61 mV×log ([K^+^]_in_/[K^+^]_out_). Independent calibration curves constructed for each mitochondrial preparation were obtained as previously described [28], [29], [31]–[33] from traces in which the extramitochondrial K^+^ level ([K^+^]_out_) was altered in the 0.1–20 mM range. The change in absorbance caused by the addition of 3 μM valinomycin was plotted against [K^+^]_out_. [K^+^]_in_ was then estimated by extrapolation of the line to the zero-uptake point [32], and the extrapolated concentration of matrix K^+^ was 130 mM, which was in close agreement with earlier observations [28]. Measurements were performed at 30°C and in a medium (pH 7.0) containing 80 mM LiCl, 50 mM HEPES, 5 mM TrisPO_4_, 1 mM EGTA and 0.1% (w/v) fatty-acid-free BSA in the presence of succinate (10 mM), rotenone (3.75 μM), oligomycin (2 μg/ml), safranin O (9.6 μM; 83.3 nmol/mg) and nigericin (80 ng/ml). Oxygen consumption and membrane potential determinations were performed by sequential additions of malonate up to 5 mM.

### Measurement of inducible leak

Mitochondrial oxygen consumption and membrane potential were measured as above in the presence of succinate (10 mM), rotenone (3.75 μM), oligomycin (2 *μ*g/ml), safranin O (9.6 μM; 83.3 nmol/mg) and 85 μM palmitate. Due to the presence of 0.1% BSA in the incubation medium, the above concentration of palmitate corresponds to 98 nM free (not bound) fatty acid, calculated using the equation described by Richieri et al. [34]. Oxygen consumption and membrane potential determinations were performed by sequential additions of malonate up to 600 μM.

### Oxidative stress parameters: mitochondrial aconitase, H_2_O_2_ release and hepatic nitrotyrosine content

To determine the aconitase-specific activity, mitochondrial aliquots were immediately frozen in liquid nitrogen and stored at −80°C. On the day of the assay, the mitochondria were sonicated and centrifuged to obtain a mitochondrial extract. The mitochondria were solubilised in 1% Triton X-100. Aconitase-specific activity was measured in a medium (pH 7.4) containing 30 mM sodium citrate, 0.6 mM MnCl_2_, 0.2 mM NADP, 50 mM Tris-HCl and 2 U of isocitrate dehydrogenase. The formation of NADPH was followed spectrophotometrically (340 nm) at 25°C. [35], [36]. The level of aconitase activity measured equals the level of active aconitase (basal level). Aconitase inhibited by ROS *in vivo* was reactivated so that total activity could be measured by incubating mitochondrial extracts in a medium containing 50 mM dithiothreitol, 0.2 mM Na_2_S and 0.2 mM ferrous ammonium sulphate [37].

To determine the H_2_O_2_ release, the final mitochondrial suspensions were maintained on ice and immediately used for the oxygen consumption and H_2_O_2_ production measurements, which were completed in less than 2 h. The rate of mitochondrial H_2_O_2_ production was assayed as previously reported [38] by measuring the increase in fluorescence (excitation at 312 nm and emission at 420 nm) caused by the oxidation of homovanillic acid by H_2_O_2_ in the presence of horseradish peroxidase. The reaction conditions were as follows: 0.25 mg of mitochondrial protein per ml, 6 U of horseradish peroxidase per ml, 0.1 mM homovanillic acid and 50 U of SOD per ml; and 5 mM succinate +0.2 μM rotenone were added as substrates at the end to initiate the reaction in the incubation buffer (pH 7.4; 145 mM KCl, 30 mM HEPES, 5 mM KH_2_PO_4_, 3 mM MgCl_2_, 0.1 mM EGTA and 0.1% albumin) at 37°C. All the assays with succinate as a substrate were performed in the presence of rotenone to avoid the backward flow of electrons to complex I [38].

To determine the hepatic nitrotyrosine content, frozen liver samples (300 mg) were homogenised in 2 ml of 25 mM HEPES (pH 7.5), 150 mM NaCl, 1% NP40, 10 mM MgCl_2_, 1 mM EDTA and 2% glycerol, followed by centrifugation. Supernatants were used for measurements of nitrotyrosine concentrations with the OxiSelect Nitrotyrosine ELISA kit (Cell Biolabs, San Diego, CA). Nitrotyrosine concentrations were normalised per mg of protein.

### Immunoblotting

Immunoblot analysis was performed as described previously [39]. The following primary antibodies were used: PPARα and β-actin (Abcam); OPA-1 (Novus Biologicals); Mfn2, DRP and Fis1 (Santa Cruz); AKT, phospho-AKT (Ser473) and UCP2 (Santa Cruz); and COX-IV (Cell Signalling Technology, Beverly, MA).

#### Data analysis

Data are expressed as the mean ± SE. Respiration rates at the highest membrane potential common to all three curves were used to test for differences in proton leak. Differences between groups were compared by ANOVA followed by the Newman-Keuls test to correct for multiple comparisons. Differences were considered statistically significant at P<0.05. All analyses were performed using GraphPad Prism 4 (GraphPad Software, San Diego, CA).

## Results

### Body weight gain and serum metabolite levels

We first analysed whether the different source of fat (lard or fish oil) in high-fat diets differentially affected obesity development and serum levels of metabolites related to obesity-linked diseases. In particular, we assessed TG, ALT, insulin and glucose levels as markers of dyslipidaemia, liver injury and insulin resistance, respectively. For obesity development, rats fed high-lard (L rats) and high-fish-oil (F rats) diets displayed similar increases in energy intake compared to the intake of rats fed standard diets (N rats), and L rats gained significantly more body weight than F rats (Table 2). Serum TG and ALT levels were each significantly higher in L rats than in N rats (Table 2), and both parameters were lower in F rats than in L rats, with TG levels not differing between F and N rats.

**Table 2:** Body weight gain, energy intake, serum parameters and hepatic parameters in rats fed a normal, HL or HFO diet.

		N	L	F
Body weight gain, g		108±10	170±9*	134±5* #
Energy intake (KJ)		13,202±400	20,132±502*	20,106±221*
Serum parameters:				
	TG, mg/dl	80.0±4.0	120.2±2.4*	84.0±3.0#
	ALT, U/l	21.0±3.6	34.8±1.5*	28.6±1.8* #
	Glucose mg/dl	83.2±2.5	107.1±5.2*	105.7±6.2*
	Insulin μg/l	0.590±0.152	1.187±0.215*	0.620±0.070#
	HOMA index	2.82±0.78	7.30±1.52*	3.76±0.31#
Liver wet weight, g		15.6±0.5	18.8±0.7*	17.6±0.8*
Hepatic lipid content, mg/g		4.1±0.2	8.5±0.3*	5.9±0.8* #

Data are means ± SE for 8 rats in each experimental group.

*P<°.05 compared to N rats, and ^#^P<0.05 compared to L rats.

N =  rats fed normal low-fat diet; L =  rats fed HL diet; F =  rats fed HFO diet.

ALT =  alanine aminotransferase.

TG =  triglycerides.

HOMA index  =  [glucose (mg/dl) × insulin (mU/l)]/405.

L and F rats displayed significantly higher serum glucose levels than N rats, whereas both the serum insulin level and the HOMA index value were highest in L rats (Table 2). Taken together, these first results showed the high fish oil feeding was associated with a lower degree of obesity development, dyslipidaemia, insulin resistance and hepatic injury compared to high lard feeding.

### Hepatic parameters

**Figure 1:**
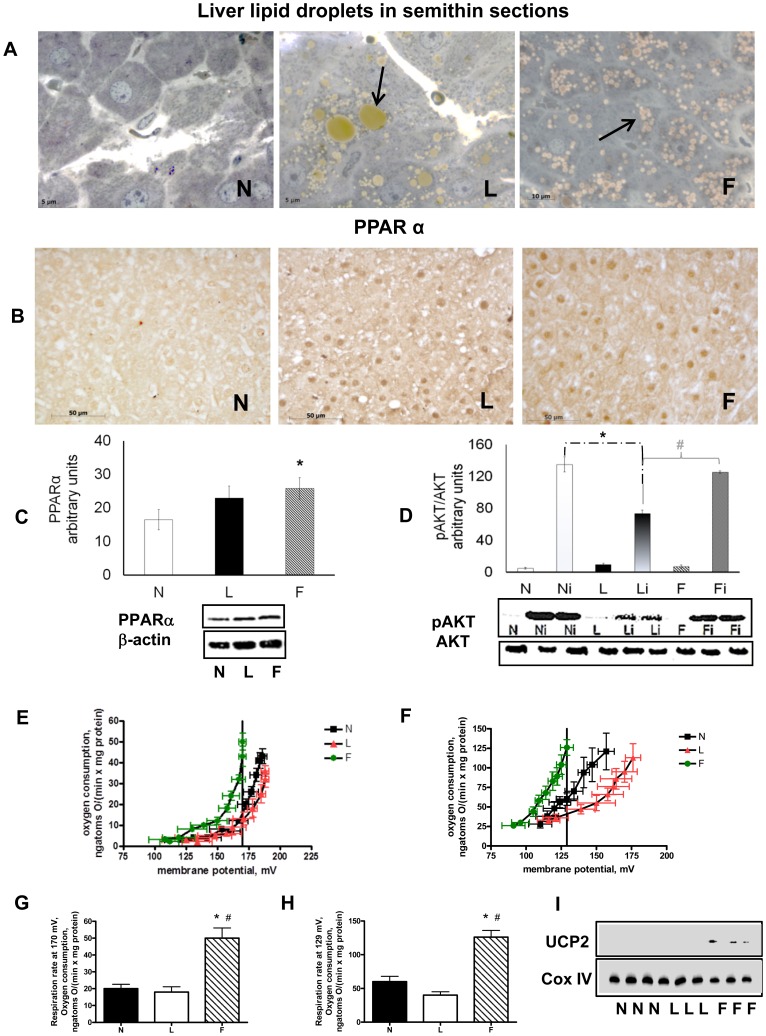
Hepatic steatosis, insulin resistance and mitochondrial efficiency. (**A**), Representative semi-thin liver sections stained with toluidine blue from N, L and F rats showing the presence and the relative abundance of lipid droplets in HF-fed rats. (**B**), Immunohistochemical reaction and (**C**), immunoblot for PPARα. The intensities of the bands were normalised to that of β-actin. (**D**), Basal and insulin-induced AKT phosphorylation. N, L, F =  sham; and Ni, Li, Fi =  insulin-injected. (**E**) and (**F**), Kinetics of basal and palmitate-induced proton leaks in isolated liver mitochondria. (**G**) and (**H**), Respiration rates measured by interpolation at 170 mV or 129 mV for basal and palmitate-induced proton leaks, respectively. Data are means ± SE for 8 rats in each group. *P<0.05 vs. N rats. #P<0.05 vs. L rats. **I**, Immunoblot for UCP2. Thin immunoreactive bands were evident only for F groups. The intensities of the bands were normalised to that of Cox IV.

We next focused our attention on hepatic parameters to further investigate the effects of both high-fat diets on hepatic steatosis and insulin-resistance development. The liver weight was higher in both L and F rats compared to control rats, and the highest value was observed in L rats (Table 2). To investigate hepatic lipid accumulation, we used both histologic observations and lipid extraction according to the method of Folch [20]. Semi-thin sections of L and F livers revealed hepatocytes moderately well-filled with lipid droplets, whereas these sections of N livers displayed little or no fat accumulation. Group L livers had both the largest lipid droplets (Figure 1A) and the highest lipid content, as determined by the Folch method (Table 2), among the three groups.

To verify whether the different degree of lipid accumulation was associated with a different degree of lipid oxidation, we analysed the presence of PPARα, a nuclear receptor implicated in the regulation of CPT, and other genes involved in mitochondrial fatty acid β-oxidation, by immunohistochemistry and western blot analysis. The results showed that PPARα immunostaining labelled the cytosol in all rat groups, whereas nuclear staining of PPARα following its activation and translocation into nuclei was more evident in F and L rats than in N rats (Figure 1B). Moreover, the PPARα content was higher in F rats than in N rats (Figure 1C).

Because the HOMA index values suggested a systemic insulin resistance in L rats, we analysed the presence and activation of AKT, a key enzyme involved in the insulin-signalling pathway, to assess the onset of insulin resistance in the liver. Basal AKT content did not differ among the three groups, but L rats exhibited the lowest value for insulin-stimulated AKT phosphorylation (at Ser473) (Figure 1D).

Taken together, these results suggested that the HFO diet did not elicit hepatic insulin resistance but that it induced a lower degree of hepatic steatosis compared to the HL diet, possibly through an ameliorated utilisation of lipid substrates mediated by PPARα.

### Liver mitochondrial function

Because mitochondria are the main site of the substrate oxidative process, we analysed respiratory rates in isolated liver mitochondria. Oxidative mitochondrial respiratory activity was determined by using both NADH- and FADH_2_-linked substrates (pyruvate plus malate and succinate plus rotenone, respectively) to involve different dehydrogenases, carriers and entry sites of reducing equivalents. We also used palmitoyl-CoA as a substrate to assess the capacity of lipid substrate oxidation.

Table 3 shows that no difference among the groups was found in the respiratory rates when pyruvate plus malate was used as a substrate both in State 3 (maximal oxidative capacity in the presence of ADP) and State 4 (in absence of ADP).

**Table 3:** Respiratory parameters and oxidative stress in liver mitochondria isolated from rats fed a normal, HL or HFO diet.

		N	L	F
Pyruvate plus malate				
	State 3	25.7±1.3	23.3±0.8	24.1±1.7
	State 4	6.5±0.4	6.1±0.7	7.1±0.3
	RCR	3.9±0.4	3.9±0.3	3.4±0.1
Succinate plus rotenone				
	State 3	200±12	153±8*	186±11#
	State 4	29.0±3.0	19.5±1.4*	26.4±1.2#
	RCR	6.9±0.6	7.8±0.6	7.1±0.5
	OCR FCCP	209.2±16.0	175.3±11.2*	190.4±13.7#
	OCR oligomycin	23.5±2.18	18.9±2.4*	26.2±2.7#
	OCR FCCP/OCR oligomycin	8.9±0.8	9.3±0.9	7.3±0.6* #
Palmitoyl-CoA plus malate				
	State 3	78.1±5.0	97.7±8.0*	121.5±3.3* #
	State 4	13.0±1.0	16.0±1.0*	17.6±1.2*
	RCR	6.0±1.0	6.1±0.6	6.9±0.8
CPT activity, nmol/min×mg protein		8.1±0.6	11.5±0.5*	14.0±0.8* #
Basal aconitase/total aconitase		0.84±0.013	0.71±0.014*	0.80±0.02#
H_2_O_2_, nmol/min×mg protein		0.208±0.02	0.261±0.001*	0.203±0.001#
pmol nitrotyrosine/mg liver protein		84.03±2.8	119.4±2.3*	78.8±4.5#

Respiratory parameters are expressed in ng atoms oxygen × min^−1^×mg^−1^ protein.

OCR =  oxygen consumption rate.

Data are means ± SE for 8 rats in each experimental group.

*P<°.05 compared to N rats, and ^#^P<0.05 compared to L rats. N =  rats fed normal low-fat diet; L =  rats fed HL diet; F =  rats fed HFO diet.

CPT =  carnitine palmitoyl transferase.

In contrast, when succinate plus rotenone was used as a substrate, both states 3 and 4 were significantly lower in L rats than in N and F rats. In addition, the fatty acid State 3 oxidation rate, which was measured using palmitoyl-CoA as a substrate, and CPT system activity were each increased (vs. N) in the L group and further increased in the F group (Table 3).

To test mitochondrial efficiency, we measured the OCR FCCP/OCR oligomycin ratio as well as basal and fatty-acid-induced proton leak kinetics. The OCR FCCP/OCR oligomycin ratio was lower in F rats than in N and L rats, suggesting reduced mitochondrial efficiency in F rats. Concerning proton leak kinetics, Figure 1E shows that under basal conditions (in the absence of free FAs), the F rats exhibited increased basal proton leak kinetics (F rat mitochondria had to consume more oxygen than either N or L rat mitochondria to maintain a given membrane potential), with N and L rats displaying superimposable kinetic curves. Moreover, at the highest membrane potential common to all three curves (170 mV), the respiration rate for F rats was significantly higher (P<0.05) than that for either N or L rats (Figure 1G).

With regard to the fatty-acid-induced proton leak (measured using physiological amounts of palmitate), L rats had the lowest proton leak among the three groups, and F rats had the highest proton leak among the three groups analysed (Figure 1F). Moreover, the respiration rates measured at the highest membrane potential common to all three curves (129 mV) were significantly different (P<0.05) among the groups (Figure 1H).

Because mild uncoupling may protect against ROS damage, mitochondrial oxidative stress markers (H_2_O_2_ production and aconitase activity) were assessed. Compared to both N and F rats, L rats exhibited increased mitochondrial ROS production based on their lower basal/total aconitase activity ratio (a sensitive marker of oxidative stress) and on their significantly higher H_2_O_2_ production in isolated mitochondria (Table 3). To test hepatic oxidative stress, nitrotyrosine content was assessed in liver homogenates. The results showed that the highest nitrotyrosine was found in L rats, confirming the higher oxidative stress found in isolated mitochondria in this group of rats compared to F and N rats.

We next tested the presence of uncoupling protein 2, which might be involved both in mitochondrial uncoupling and ROS protection. Western blot analysis showed that UCP2 was undetectable in N and L rats, but a thin UCP2 band was observed in F rats (Figure 1 I).

### Electron microscopy (EM)

**Figure 2:**
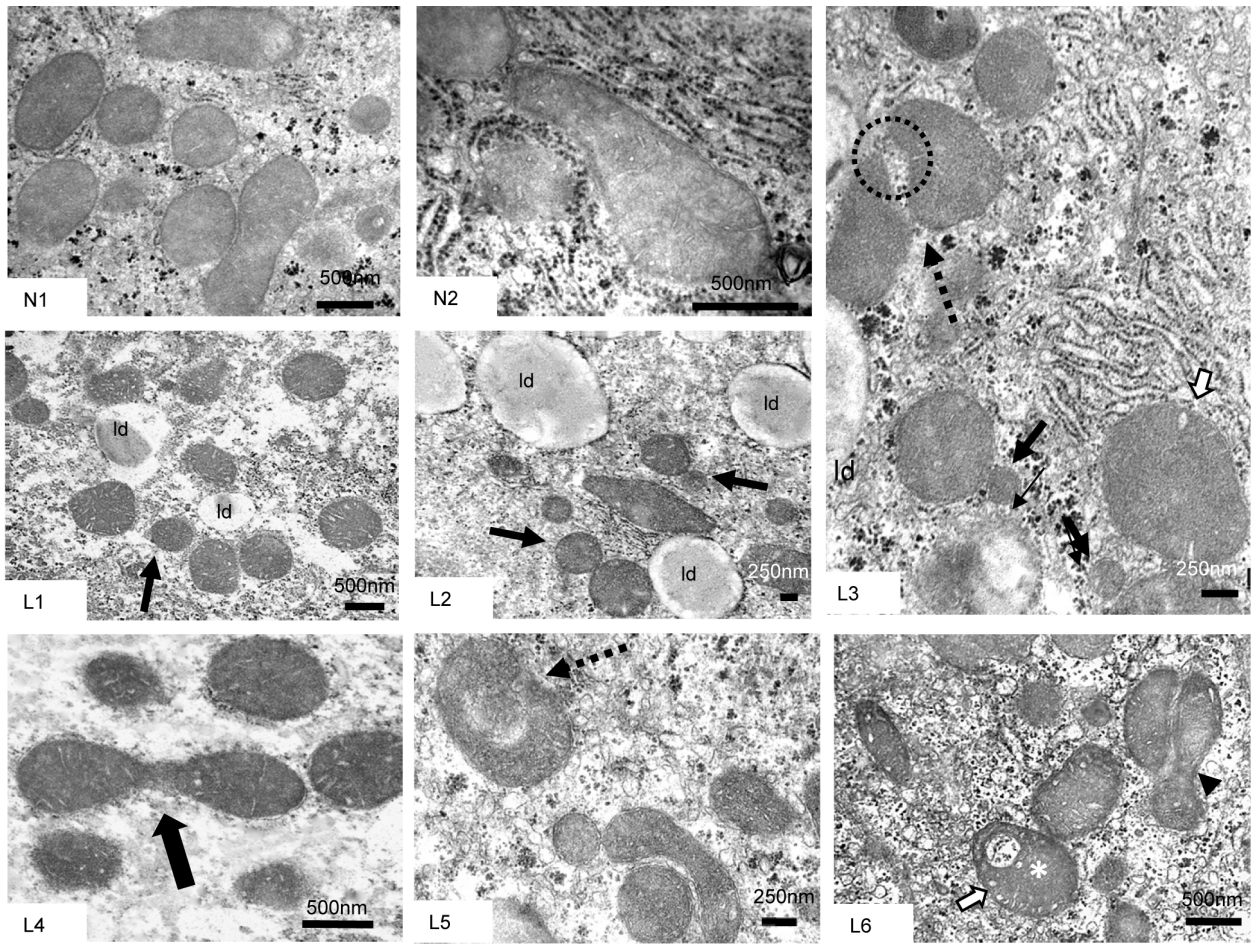
N and L rat mitochondrial features. (N1–N2), N mitochondria with lamellar cristae and moderately dense matrix. (L1–L6), L mitochondria exhibited a dense matrix, and a few appeared slightly swollen (white arrows). Small, round mitochondria (L1) were often observed close to others (L2, L3, black arrows). (L3), Two mitochondria coming into contact at two sites (interrupted arrow). (L1–L3), Lipid droplets (ld) close to mitochondria. (L4) Dumbbell-shaped mitochondrion, suggesting on-going fission (black arrow). (L5) Cup-shaped mitochondrion enveloping another mitochondrion (interrupted arrow), suggesting a donut formation. (L6) Asymmetric dumbbell-like mitochondrion close to another mitochondrion (arrowhead) as well as a donut-like mitochondrion (white asterisk).

Because changes in mitochondrial function may be associated with changes in mitochondrial morphology, EM observations were performed to evaluate ultrastructural features in the three groups of experimental rats. Mitochondria in N rats displayed a moderately electron-dense matrix, lamellar cristae and regular boundaries (Figure 2 N1-2).

Mitochondria in L rats mostly exhibited an electron-dense matrix, with the mitochondria appearing slightly swollen in some cases, and the intracristal spaces were often larger than those observed in control preparations. The presence of numerous small, round mitochondria (often in contact with lipid droplets) (Figure 2 L1-3) and mitochondria with a dumb-bell shape suggested division processes (Figure 2 L4). In contrast, we observed both the presence and the apparent formation of donut-like mitochondria by autofusion and anomalous fusion between two organelles, as suggested by comma-shaped or ring-like mitochondria in some instances enveloping other mitochondria. We inferred that when the adjacent ends of mitochondria come into contact, a hole enclosing cytoplasm may be created (Figure 2 L3, L 5–6).

**Figure 3:**
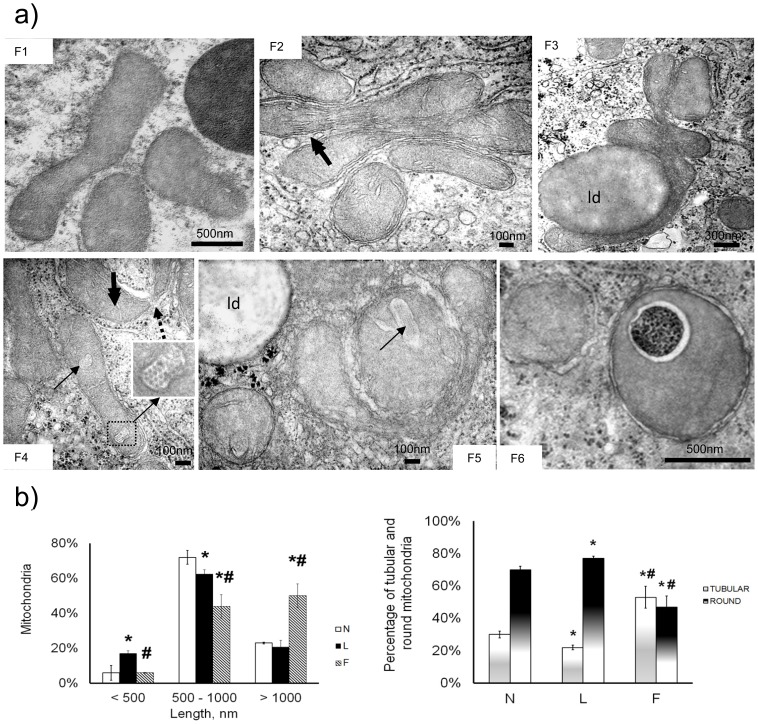
F rat mitochondrial features. Mitochondrial length and tubular/round ratio. (**A**), F1–6, F mitochondria exhibited light matrix and numerous cristae parallel with the longitudinal axis (double-arrow). F1: Boomerang-shaped mitochondria, suggesting a previous fusion process. F2–3: Mitochondrial cluster forming a fused network. F3: Long, cup-shaped mitochondrion surrounding a lipid droplet (ld). F4–5: Paracrystalline inclusions (black arrows) in the intracristal space. Donut-shaped formation (interrupted arrow). F6: Autophagosome containing a donut-like mitochondrion with enclosed degraded cytoplasm. (**B**) and (**C**), Based on EM images, L rats had the shortest mitochondria and the lowest tubular/round ratio (%). *P<0.05 vs. N. #P<0.05 vs. L rats.

Mitochondria in F rats exhibited regular cristae and a less electron-dense matrix than that of L rat mitochondria. The presence of boomerang-shaped mitochondria and several mitochondria clustered together suggested fusion and network formation in F rats (Figure 3A F1–2). The clustered mitochondria often surrounded lipid droplets (Figure 3A F3). In some mitochondria in F and L rats, dilated intracristal spaces encased ordered parallel aggregates of helical filaments, which appeared as a close hexagonal array of microcylinders in cross-section (Figure 3A F4–5). Donut-like mitochondria were also found in F livers. Figure 3A F6 depicts one such mitochondrion enveloped by a lysosomal membrane.

To examine the distribution of mitochondrial length, we divided liver mitochondria into three length ranges, which revealed that L and F rats exhibited the highest percentages of short and long mitochondria, respectively (Figure 3B). Furthermore, most of the mitochondria in L rats were round, whereas round and tubular mitochondria were almost equally represented in F rats (Figure 3C). Because these observations suggested a different distribution of mitochondrial length among the three groups of rats, we next assessed mitochondrial dynamic behaviour, in which an imbalance has been suggested to be involved in obesity and insulin resistance. In particular, we evaluated the presence of the proteins relevant to mitochondrial dynamics by western blot analysis and immunohistochemistry.

### Proteins relevant to mitochondrial dynamics

**Figure 4:**
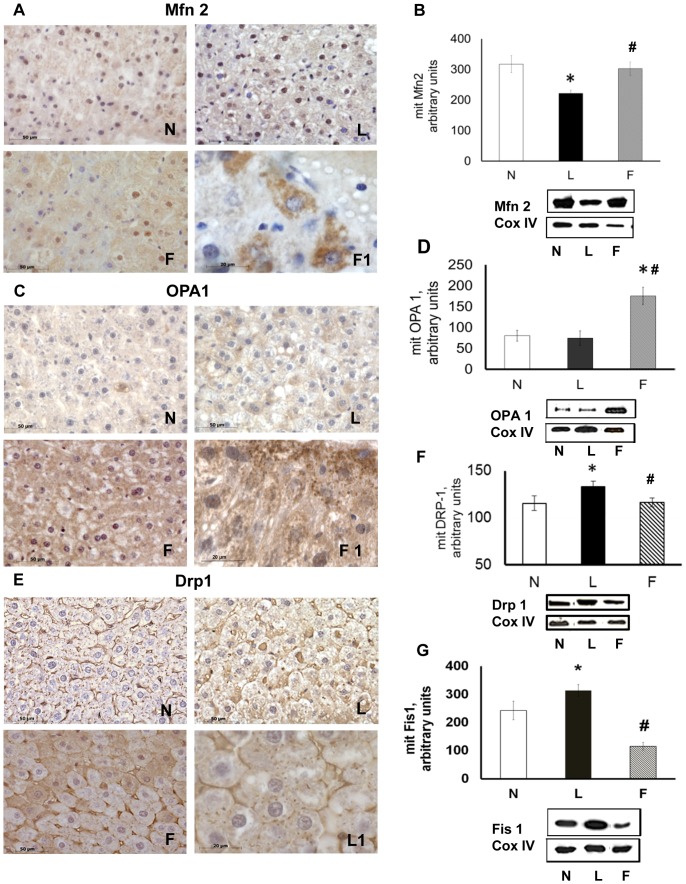
Proteins relevant to mitochondrial dynamics. (**A**), Immunohistochemical reaction and (**B**), immunoblot for Mfn2. (**C**), Immunohistochemical reaction and (**D**), immunoblot for OPA1. (**E**), Immunohistochemical reaction and (**F**), immunoblot for Drp1. (**G**), Immunoblot for Fis1. (**B**), (**D**), (**F**) and (**G**), Densitometric analysis data shown as means ± SE. *P<0.05 vs. N rats. #P<0.05 vs. L rats. For all immunoblots, representative blots are shown for each protein of interest. The intensities of the bands were normalised to that of Cox IV.

Immunostaining of N rat liver sections for Mfn2 revealed numerous positive hepatocytes. In F rat liver sections, Mfn2 labelling was even more evident, with some strongly immunoreactive cells, but Mfn2 labelling was weak in L rat livers (Figure 4A). Moreover, L rat livers had the lowest Mfn2 content in mitochondrial extracts as measured by western blot analysis (Figure 4B).

OPA1 immunostaining labelled most of the F rat liver mitochondria, but such staining in N and L rat livers was weak (Figure 4C). F rats exhibited significantly higher OPA1 content than either N or L rats (Figure 4D).

In N rat livers, Drp1 immunostaining was localised to numerous mitochondria (Figure 4E N), but in F rat livers, mitochondria in only a few cells were labelled (Figure 4E F). In L rat livers, Drp1 immunostaining heavily labelled the cytoplasm and mitochondria, which appeared fragmented to various degrees (Figure 4E L–L1). Moreover, L rats had the highest Drp1 content in their mitochondrial extracts (Figure 4F).

The Fis1 mitochondrial content was highest in L rats and lowest in F rats (Figure 4G). Together, these results suggested a shift towards fission processes in L rats and a shift towards fusion processes in F rats.

## Discussion

The current study serves to focus attention on functional and structural features that could be differentially impacted by high-lard and high-fish oil feeding. The high-fish oil feeding elicited a lower degree of obesity development and IR compared to high-lard feeding, in accordance with previous studies suggesting that omega-3 fatty acids have anti-obesity effect and potential benefits for IR [16], [18]. In our results, L and F rats showed blood glucose levels higher than N rats, but within the normal range [40], [41]. It should be noted that F rats showed higher blood glucose level vs. N rats, but normal insulin level, whereas L rats exhibited both higher blood glucose and insulin values vs. N rats. It has been reported that the composition of dietary fat directly influences glucose-stimulated insulin secretion [42], in fact dietary saturated fatty acids stimulate insulin secretion in rats [43], whereas long-chain omega-3 fatty acids decrease insulin secretion leading to a ‘diabetogenic effect’ [43]. In particular, measurement of insulin release from pancreas of fasted rats perfused with glucose and a range of individual fatty acids of varying degree of saturation revealed that the fold stimulation of insulin secretion was greater for saturated vs. unsaturated fatty acids [44]. It has been suggested that omega-3 fatty acids act directly at the level of the β-cell to modify insulin responsiveness to glucose. [45]. Therefore, a reduced responsiveness of pancreatic β-cells to plasma glucose elevation and a reduced suppression of hepatic glucose output by insulin may contribute to the rise in fasting glucose observed in our F rats which exhibited normal insulin levels.

We then focused our attention on the effects of the two different fat dietary sources in liver and the results revealed that the HL diet induced fat accumulation and IR, which was parallel to impaired mitochondrial function, and a dysregulated expression profile of proteins participating in mitochondrial dynamics. In contrast, the HFO diet led to less hepatic lipid accumulation through improved mitochondrial fatty acid utilisation supported by a shift towards fusion, which counteracted oxidative stress and possibly the hepatocyte damage induced by long-term over-feeding. This study suggests an association between mitochondrial dynamic behaviour and functionality in the liver in rats fed a high-fat diet, and it also suggests that both dynamics and functions are affected differently between diets rich in lard or in fish oil.

Hepatic mitochondrial respiratory capacity was determined by measuring State 3 and 4 respiration with various substrates to involve different dehydrogenases, carriers and entry sites of reducing equivalents into the respiratory chain. With regard to the respiration rates measured in the presence of a NADH-linked substrate, namely, pyruvate plus malate, no significant differences were found among the experimental groups. In contrast, when succinate was used as the FADH_2_-linked substrate, a significant decrease in State 3 respiration was observed in L rat mitochondria. In addition, when a lipid substrate (palmitoyl CoA) was used, L rat mitochondria showed significantly higher State 3 respiration compared to control mitochondria. It should be noted that a decrease in succinate State 3 respiration may be due to defects both in the activity of substrate oxidation reactions (which are complex II, complex III, complex IV and dicarboxylate carrier in this case) and/or in the activity of the phosphorylation reactions (ANT, ATP synthase and phosphate carrier). The results obtained with succinate plus FCCP (a condition in which the oxidation reactions are close to the State 3 kinetic rate but the phosphorylating reactions no longer exert control over respiration) appeared to exclude phosphorylating reactions from being the site of impairment. In fact, the data showed that the impairment in oxygen consumption remained, with a significant decrease in FCCP respiration in L rat mitochondria, suggesting that the defect resided in the substrate oxidation reactions and not in phosphorylating reactions. Many of the reactions controlling State 3 respiration with succinate also participate in State 3 respiration with pyruvate or palmitoyl-CoA. The fact that the State 3 rate with malate/pyruvate in L rat mitochondria was not different from that in N rat mitochondria and was similar to the State 4 (and oligomycin) succinate rate may indicate that the defect in the succinate State 3 rate resided within complex II (rather than complex III or IV).

However, the lower succinate State 4 respiration in L rat mitochondria may not necessarily result from a complex II defect; Figure 1E/F illustrates the lower proton leak in L rat mitochondria, which might account for the difference in State 4 respiration because proton leak is a major controller of State 4 (the difference shifts to a complex II defect as the mitochondria approach State 3).

The increased capacity of mitochondria to oxidise acyl-CoA in L rats might serve as a compensatory mechanism for the elevated hepatic fatty acids uptake that occurs during high fat feeding, which is in accordance with previous results [4], [5]. The observed increases in PPAR-α nuclear immunostaining and CPT system activity may be relevant to this enhanced β-oxidation. However, this increase in acyl-CoA oxidation capacity was not sufficient to handle the increased load of hepatic free FAs, resulting in lipid accumulation in L rat livers. With the limitation that *in vitro* differences may not translate to *in situ* differences, the fat accumulation was most likely also due to an increase in mitochondrial energy efficiency, as shown by the decrease in induced proton leak. The presence of an increase in β-oxidation rate [46], [47] might play a crucial role in enhancing ROS production, as suggested by the increased H_2_O_2_ production, inhibited aconitase activity in L rat mitochondria and increased nitrotyrosine content in L rat liver homogenates. However, it should be noted that H_2_O_2_ production measurements were performed in the presence of succinate and not in the presence of palmitoyl CoA, which is critical because different substrates can substantially affect the rates of H_2_O_2_ production. Moreover, the measured H_2_O_2_ production rates were under State 4, non–phosphorylating conditions, whereas *in situ* the actual rate of production can vary substantially depending on the overall rate of cellular ATP consumption.

By comparison with the HL diet, the HFO diet led to less lipid accumulation associated with greater increases in PPAR-α content, CPT system activity and β-oxidation rate. Moreover, in the HFO rats, the decreased mitochondrial energy efficiency, as evidenced by the increased basal and induced proton leaks, might also have contributed to increased fatty acid utilisation. The mild uncoupling observed in F rats may have been due to an enhanced expression of uncoupling protein 2 (UCP2). To test this hypothesis, we analysed UCP2 content, and we found a detectable amount of UCP2 protein only in F rat mitochondria, which was in accordance with previous results showing increased UCP2 expression induced by PUFAs [48]. We cannot exclude the possibility that the mild uncoupling found in this work may be mediated by anion carriers (such as ADP/ATP antiporter) or by changes in internal mitochondrial membrane fluidity [49], [50]. However, this uncoupling by preventing respiratory complex over-reduction might serve to counteract excessive ROS formation [51], which was supported by F rats exhibiting a significantly lower mitochondrial H_2_O_2_ release, higher mitochondrial basal aconitase/total aconitase ratio and lower liver homogenate nitrotyrosine content compared to L rats. This functional adaptation in mitochondria (through a mild uncoupling that reduced ROS production) may protect against the initial damage induced by a high-fat diet, which may lead to hepatosteatosis and thus may be an adaptation similar to that found in A/J mice, which are resistant to high-fat-diet-induced hepatosteatosis and obesity [52]. It should be noted that F rat mitochondria, even with a higher proton leak, did not exhibit significantly lower H_2_O_2_ production rates than N rat mitochondria because a greater supply of lipid substrates reached the liver due to hyperlipidic feeding, leading to an increase in lipid oxidation. This increase should have led to a greater ROS production, but this trend was counteracted by increasing proton leak, thereby maintaining normal H_2_O_2_ levels. However, this adaptation was not able to further reduce H_2_O_2_ levels below normal values. Further studies are needed to test this hypothesis by using more physiological substrates, such as palmitoyl CoA, in the measurements of H_2_O_2_ production.

Concerning morphological features, the EM observations revealed certain similarities but many different morphological adaptations between L and F rats. A peculiar feature observed in both L and F rats was the presence of paracrystalline inclusions. The role of paracrystalline inclusions has not yet been clarified. Paracrystalline inclusions may be a normal constituent of mitochondria, or they may reflect either mitochondrial injury (e.g., NASH patients exhibit paracrystalline inclusions in mega-mitochondria) or an adaptive process [6].

The observed mitochondrial dynamic behaviour differed between L and F rats. In L rats, the expression of mitochondrial fission proteins was enhanced and the expression of fusion proteins was reduced, suggesting an imbalance towards mitochondrial fragmentation, which agreed with the observation of smaller mitochondria, with round mitochondria being more prevalent that tubular ones in ultra-thin sections from L rats. In contrast, the expression of mitochondrial Mfn2 and Drp1 in F rats was closer to that observed in the control group. However, the Opa1 levels and Fis1 levels were significantly higher and lower, respectively, in F rats compared to N rats, supporting the shift to elongated mitochondria observed in the EM analysis.

Regarding the proteins involved in fusion/fission processes, the L group experienced a reduced expression of Mfn2 and an increase in the mitochondrial fission proteins, Drp1 and Fis1, which was consistent with the down-regulation of Mfn2 reported in skeletal muscle in obese insulin-resistant humans and in obese rodents [9], [12], [53]. Recent data have indicated that hepatic ablation of Mfn2 causes glucose intolerance and deficient hepatic insulin signalling [54]. Based on these data, we propose that the reduced Mfn2 expression upon exposure to the HL diet participates in the insulin resistance that affects the liver under these conditions.

Moreover, mitochondrial fragmentation and enhancements of fission machinery have been reported after treatment with high glucose (HG) and/or high free FAs in the following models: insulin-resistant 3T3-L1 adipocytes (which have smaller and more compact mitochondria) [55], pancreatic islet cells exposed to glucose and fatty acid [56] and skeletal muscle in genetically obese and diet-induced obese mice [14]. Interestingly, upon exposure to HG concentrations, mitochondrial fragmentation has been shown to occur in association with ROS formation in both a rat liver cell line and myoblasts [57]. These authors suggested that the small spherical mitochondria formed in HG conditions may represent condensed, metabolically active organelles with increased total surface area, which is a modification that would increase the accessibility of metabolic substrate to carrier proteins. Such fragmentation might induce alterations in electron transport and coupling, leading to an increase in ROS [10], [57]. Therefore, in L rat livers, mitochondrial fragmentation may be an adaptive cellular response to increases in the mitochondrial intake and oxidation of FAs, which would result in elevated ROS production.

Compared to the HL diet, the HFO diet led to a mild uncoupling with less ROS production as well as a shift towards fusion (with concomitant increases in Mfn2 and OPA1 as well as decreases in Drp1 and Fis1) together with an improvement in insulin sensitivity and to less lipid accumulation. This result suggests that the high caloric intake *per se* is not the key factor involved, and the specific fatty acid composition may be relevant to consider. The different expression pattern of mitochondrial dynamic proteins when comparing the F and L groups was consistent with the observation that the inhibition of mitochondrial fission ameliorates muscle insulin signalling and systemic insulin sensitivity in obese mice [14] as well as the observation that Mfn2 deficiency impairs insulin signalling in both mouse liver and muscle [54]. Moreover, a previous study reported mitochondrial fission during *in vitro* hepatocyte steatosis and its reversal by omega-3 fatty acids (i.e., tubular elongation and Mfn2 upregulation) [58]. Therefore, our present results confirmed hepatic mitochondrial fusion through fish oil *in vivo*. The mechanism underlying fish oil/omega-3 fatty acid mitochondrial fusion stimulation may involve receptor-mediated signalling and/or lipid composition, among other factors. Further studies are needed to elucidate this mechanism.

An interesting phenomenon that may be linked to the fusion/fission balance was the presence of donut-like mitochondria in both L and F rats. Donut-like mitochondria may result from anomalous fusion activity during metabolic stress both when the fission/fusion machinery is heavily tilted towards fusion [59] and when it is tilted towards fission [60]. Such a condition may be transient and followed by recovery to linear mitochondria or, in some instances, by mitophagy, as shown in Figure 2 F6. The presence of donut-like mitochondria in both L and F rat livers suggested that they may represent a mitochondrial adaptation to the stress induced by high fat feeding [61], either leading to the elimination of impaired organelles or aiding functional recovery.

In conclusion, the results of the present work suggested that the HL diet induced hepatic fat accumulation and IR, with an associated impairment of mitochondrial function, fission-shifted dynamic behaviour and oxidative stress, and that the HFO diet led to less hepatic lipid accumulation through improved mitochondrial fatty acid utilisation, which was concomitant with a fusion phenotype similar to that of the controls, thereby counteracting oxidative stress and possibly the hepatocyte damage induced by long-term over-feeding. To support these suggestions, we are planning further experiments to analyse whether the genetic manipulation of specific proteins participating in mitochondrial dynamics rescues the phenotype observed in response to a high-lard diet.
