# Expression of Concern: High-Lard and High-Fish-Oil Diets Differ in Their Effects on Function and Dynamic Behaviour of Rat Hepatic Mitochondria

**DOI:** 10.1371/journal.pone.0265521

**Published:** 2022-03-16

**Authors:** The PLOS ONE Editors

After this article [1] was published, concerns were raised about western blot results. Specifically,

- Fig 1D, AKT blot: Similarities were noted between data shown in lanes 7 and 9, between lanes 1 and 3, and between lanes 2 and 6 (flipped horizontally).
- Fig 4F: There appear to be vertical discontinuities between lanes 1/2 and lanes 2/3 of each western blot panel.
- Figs 1C, 1D, 1I, 4B, 4D, 4F and 4G: The image quality and/or brightness-contrast levels in the western blot panels do not allow a clear assessment as to the integrity or background information within the images as the background signal across each of the blots is quite uniform.

**Fig 1:**
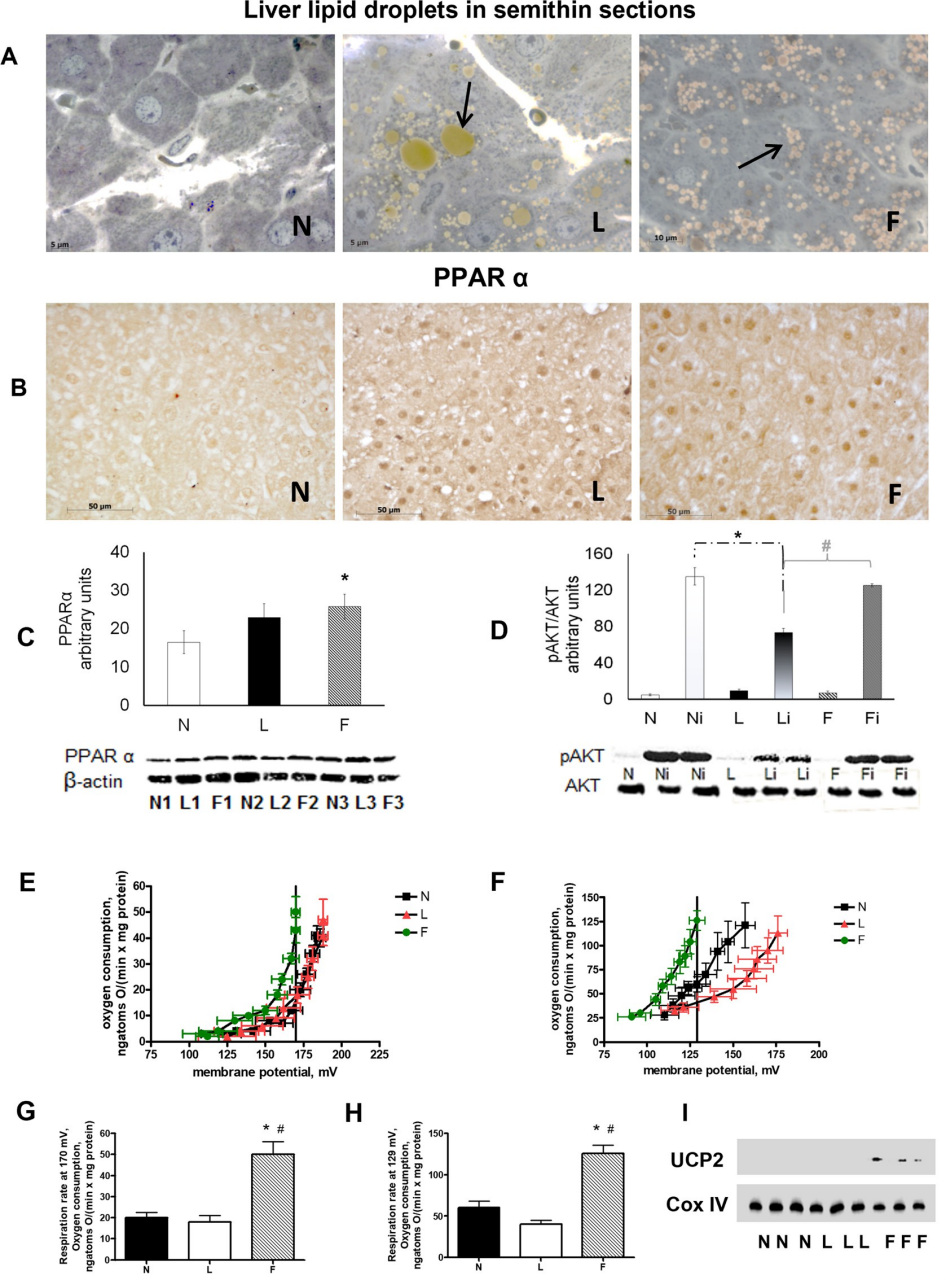
Hepatic steatosis, insulin resistance and mitochondrial efficiency. (**A**), Representative semi-thin liver sections stained with toluidine blue from N, L and F rats showing the presence and the relative abundance of lipid droplets in HF-fed rats. (**B**), Immunohistochemical reaction and (**C**), immunoblot for PPARα. The intensities of the bands were normalised to that of β-actin. (**D**), Basal and insulin-induced AKT phosphorylation. N, L, F  =   sham; and Ni, Li, Fi  =   insulin-injected. (**E**) and (**F**), Kinetics of basal and palmitate-induced proton leaks in isolated liver mitochondria. (**G**) and (**H**), Respiration rates measured by interpolation at 170 mV or 129 mV for basal and palmitate-induced proton leaks, respectively. Data are means ± SE for 8 rats in each group. *P<0.05 vs. N rats. #P<0.05 vs. L rats. (**I**), Immunoblot for UCP2. Thin immunoreactive bands were evident only for F groups. The intensities of the bands were normalised to that of Cox IV.

**Fig 4:**
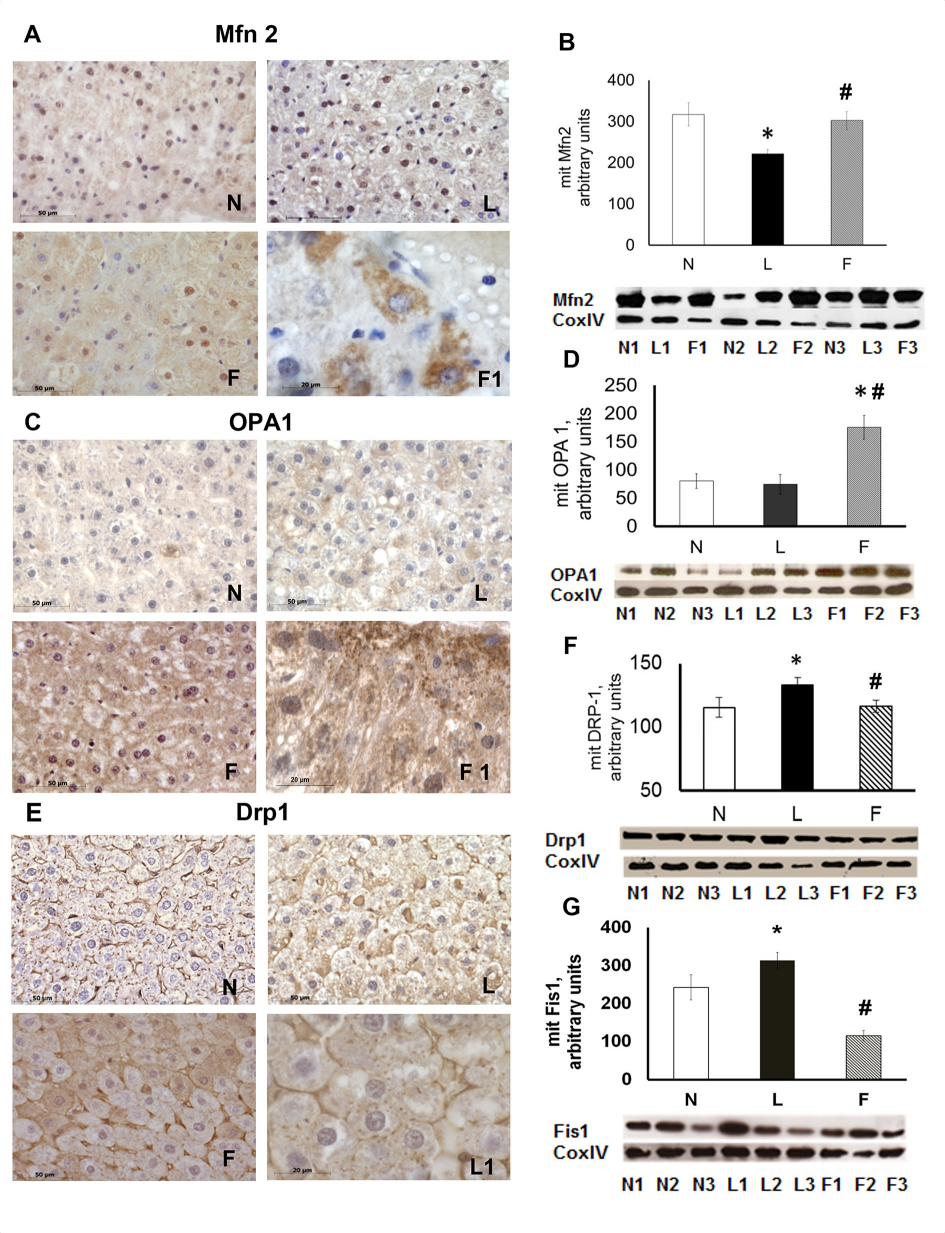
Proteins relevant to mitochondrial dynamics. (**A**), Immunohistochemical reaction and (**B**), immunoblot for Mfn2. (**C**), Immunohistochemical reaction and (**D**), immunoblot for OPA1. (**E**), Immunohistochemical reaction and (**F**), immunoblot for Drp1. (**G**), Immunoblot for Fis1. (**B**), (**D**), (**F**) and (**G**), Densitometric analysis data shown as means ± SE. *P<0.05 vs. N rats. #P<0.05 vs. L rats. For all immunoblots, representative blots are shown for each protein of interest. The intensities of the bands were normalised to that of Cox IV.

The corresponding author stated that the original data underlying results reported in this article are no longer available.

The corresponding author further explained that densitometric analysis was performed on scans of the original western blot films/plates, after which representative blot images were selected for the figures. In preparing figures, images were adjusted for brightness/contrast, and in some cases image fragments were rotated or re-ordered to present bands at a consistent orientation and layout across the panel. The corresponding author stated that all data in each panel originated from the same blot, and adjustments to brightness and contrast were applied to all data consistently in the assembled figures.

In regard to the concerns in Fig 1D, the corresponding author confirmed there are similarities across lanes in the AKT panel, but asserted that the data in the indicated lanes are different. Without the original blot image this issue remains unresolved. The corresponding author provides here another version of Fig 1 that includes more background area for the western blot panels in Fig 1C and 1D compared to the published version, and in which image splicing in panel D is visible. The corresponding author stated that image splicing occurred between lanes 3 and 4 and between lanes 6 and 7 in the AKT blot shown in panel D to present samples in an order matching the presentation in other figures. The corresponding author also provided replication data in support of Fig 1D (S1 and S2 Files). A member of *PLOS ONE*’s Editorial Board assessed S1 and S2 and stated that the methods are appropriate and the data support the findings of Fig 1D.

The corresponding author provides here an alternative version of Fig 4 which they stated includes all experimental lanes from the original blots without splicing, rearrangement or brightness/contrast adjustments. Although only cropped images are available, the blots presented in this alternative version clarify where lanes were rearranged in preparing the published versions of Fig 4D, 4F and 4G.

The corresponding author provides results of real-time quantitative PCR (RT-qPCR) for UCP2 mRNA (S3 and S4 Files) to support the results of the western blot for UCP2 protein levels in Fig 1I. The author stated that the mRNA used for RT-qPCR was collected under the same experimental conditions as those described in Fig 1I. The corresponding author acknowledges Vincenzo Migliaccio (Department of Chemistry and Biology “Adolfo Zambelli”, University of Salerno, Italy) who is not a listed author on [1] for generating these data. A member of *PLOS ONE*’s Editorial Board assessed S3 and S4, and stated that assessment of mRNA expression by RT-qPCR are not sufficient to reproduce the findings shown in the western blot in Fig 1I as a change in mRNA expression does not necessarily correspond to a change in protein expression.

In light of the unresolved concerns in Fig 1D and the unavailability of data to support results reported in this article, the *PLOS ONE* Editors issue this Expression of Concern to notify readers of the above concerns and relay the supporting data and updated figures provided by the corresponding author.

## Supporting information

S1 File Replication of the western blot experiment presented in Fig 1D. pAKT/AKT western blotting analysis was performed following the protocol for gel electrophoresis and transfer described in [1] and using 12% SDS-PAGE gel. For each sample, 50 μg of proteins from total liver homogenate were loaded, and the following antibodies and conditions were used: AKT, Cell signalling (cat. #9272), rabbit, 1:500 in 5% milk-TBS-Tween (incubation O.N. 4°C); pAKT (S473), Cell signalling (cat. #9271S), rabbit, 1:500 in 2% BSA-TBS-Tween (incubation O.N. 4°C); Anti-rabbit secondary antibody, Santa Cruz (sc-2004), 1:2000 in 5% milk 1X TBS-Tween 1% (incubation 1h, RT). N, treated with the standard laboratory diet; L, treated with the High-Lard diet; F, High-Fish oil diet; Ni, Li and Fi, treated with the previously-mentioned diets and stimulated with insulin.
(TIF)

S2 File Raw data and quantification of western blot data presented in S1 File. (XLSX)

S3 File Real-time quantitative PCR assessment of UCP2 mRNA. RT-q-PCR for UCP2 was performed using samples from 4 different animals for each experimental group (N, L and F). Total RNA was extracted from liver fragments processed according to the Tri-Reagent (Sigma-Aldrich) protocol. After extraction, 1μg of total RNA was processed with QuantiTect Reverse Transcription Kit (Qiagen) according to the manufacturer’s protocol to remove genomic DNA contamination and for the subsequent cDNA synthesis. The analyses were carried out on an Applied Biosystem 7500 Real-Time PCR System, using SYBR Green method (Life Technologies), following the procedures recommended by the manufacturer. Reactions were performed using forward and reverse primers designed using Primer Express software (Applied Biosystems). Each amplification mixture of 20 μl final volume contained 12 μl of real-time PCR Master Mix, 1 μl each of UCP2 or β-actin forward and reverse primers (10 μM), 2 μl of cDNA diluted 1:1 and 4 μl of nuclease-free water. Amplifications were performed with an initial step at 95°C for 1 min, followed by 40 cycles at 95°C for 15 s and 60°C for 40 s. A melting curve analysis of PCR products was performed from 60°C to 95°C in order to ensure gene specific amplification. UCP2 gene amplification was associated with β-actin as standard control. Changes in the UCP2 gene expression in the different samples were obtained in according to standard 2^−ΔΔCt^ method [2]. UCP2 primer sequences: forward primer, 5’-AGCAGTTCTACACCAAGGGC-3’; reverse primer, 5’-AGAGGTCCCTTTCCAGAGGC-3.’ β-actin primer sequences: forward primer, 5’-ACCCGCCACCAGTTCGCCAT-3’; reverse primer, 5’-CGGCCCACGATGGAGGGGAA -3’. Specificity of UCP2 and B-actin primers was tested with conventional PCR that retrieved a single cDNA fragment of 230 bp and 128bp, respectively. The UCP2 cDNA fragment was also sequenced using automated methods on an ABI PRISM Genetic Analyzer (PE Biosystems). The results showed 100% homology with the Rattus norvegicus UCP2 gene.
(TIF)

S4 File Raw data and quantification of RT-qPCR data presented in S2 File. (XLSX)
