# Supplementary figures and images for: Expression of Concern: High-Lard and High-Fish-Oil Diets Differ in Their Effects on Function and Dynamic Behaviour of Rat Hepatic Mitochondria
Source: PLoS One. 2022 Mar 16;17(3):e0265521. doi: 10.1371/journal.pone.0265521 (PMC8926271; doi:10.1371/journal.pone.0265521)

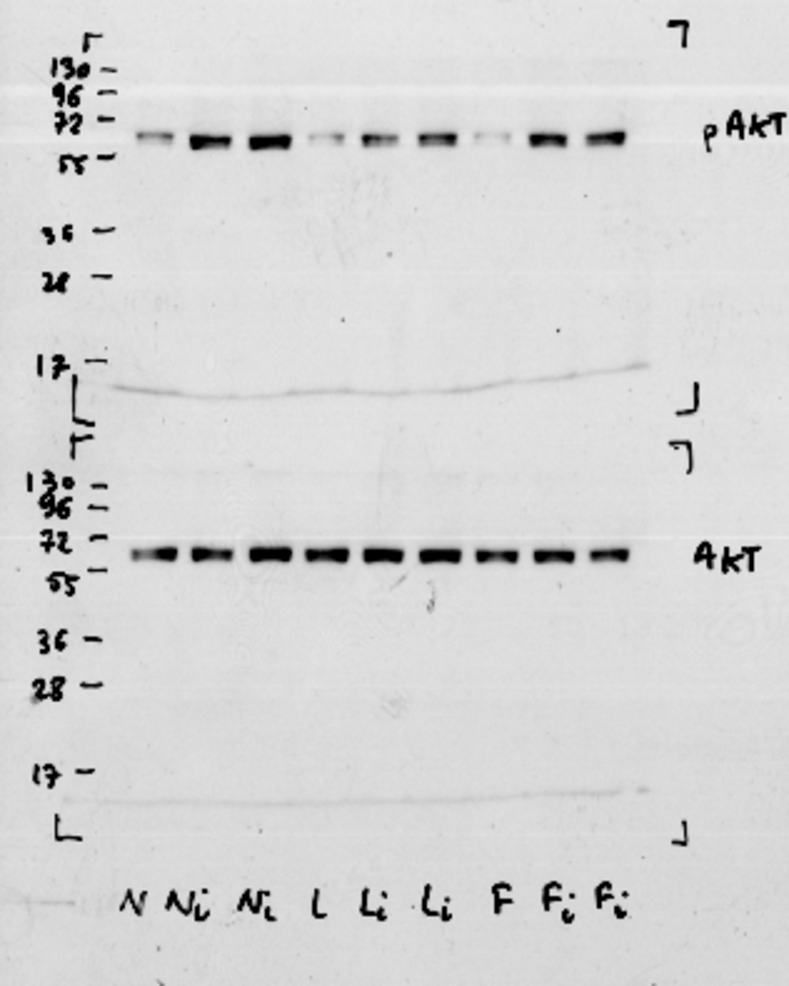

Supplement: S1 File — pAKT/AKT western blotting analysis was performed following the protocol for gel electrophoresis and transfer described in [1] and using 12% SDS-PAGE gel. For each sample, 50 μg of proteins from total liver homogenate were loaded, and the following antibodies and conditions were used: AKT, Cell signalling (cat. #9272), rabbit, 1:500 in 5% milk-TBS-Tween (incubation O.N. 4°C); pAKT (S473), Cell signalling (cat. #9271S), rabbit, 1:500 in 2% BSA-TBS-Tween (incubation O.N. 4°C); Anti-rabbit secondary antibody, Santa Cruz (sc-2004), 1:2000 in 5% milk 1X TBS-Tween 1% (incubation 1h, RT). N, treated with the standard laboratory diet; L, treated with the High-Lard diet; F, High-Fish oil diet; Ni, Li and Fi, treated with the previously-mentioned diets and stimulated with insulin. (TIF) [file pone.0265521.s001.tif]

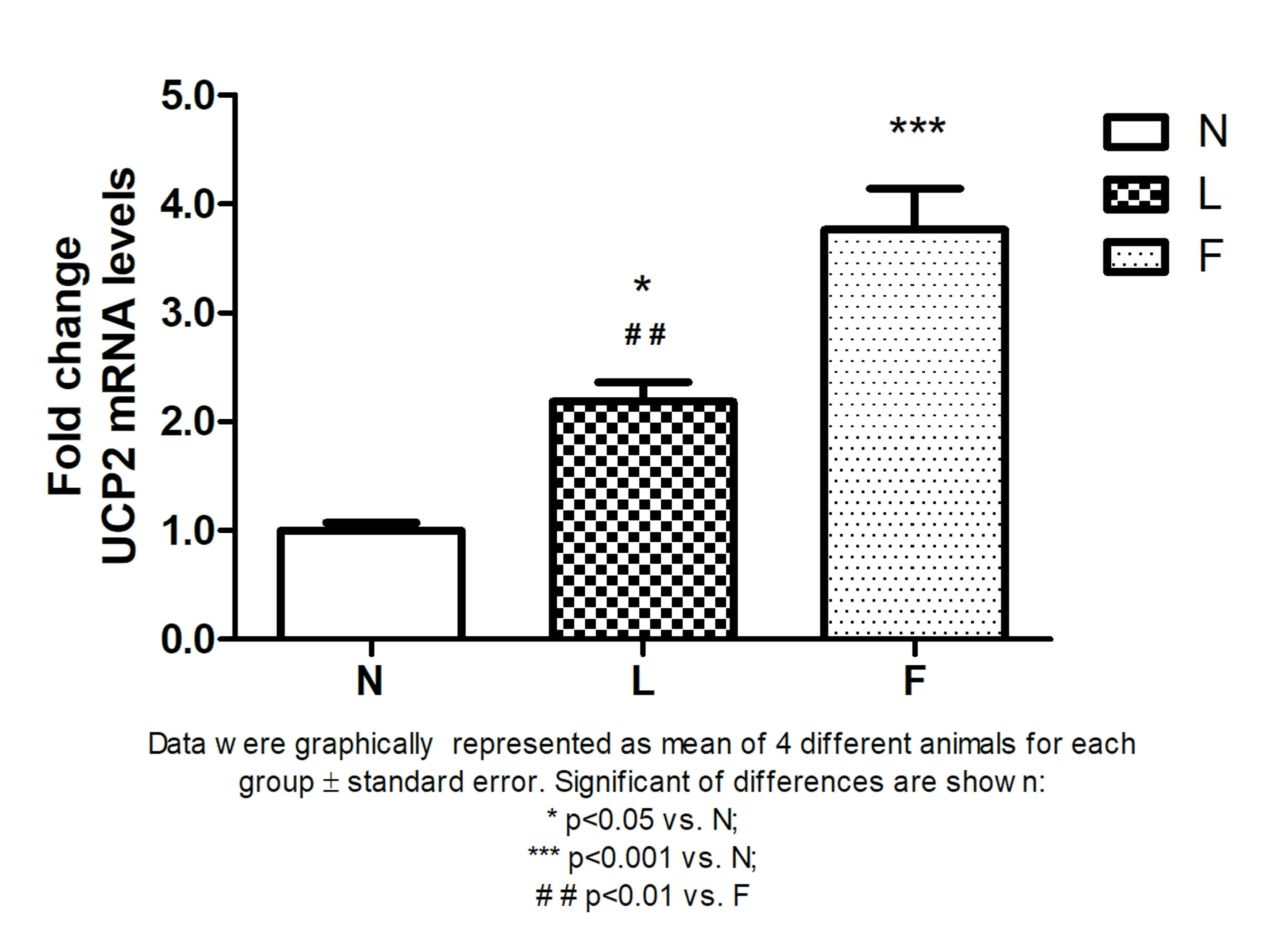

Supplement: S3 File — RT-q-PCR for UCP2 was performed using samples from 4 different animals for each experimental group (N, L and F). Total RNA was extracted from liver fragments processed according to the Tri-Reagent (Sigma-Aldrich) protocol. After extraction, 1μg of total RNA was processed with QuantiTect Reverse Transcription Kit (Qiagen) according to the manufacturer’s protocol to remove genomic DNA contamination and for the subsequent cDNA synthesis. The analyses were carried out on an Applied Biosystem 7500 Real-Time PCR System, using SYBR Green method (Life Technologies), following the procedures recommended by the manufacturer. Reactions were performed using forward and reverse primers designed using Primer Express software (Applied Biosystems). Each amplification mixture of 20 μl final volume contained 12 μl of real-time PCR Master Mix, 1 μl each of UCP2 or β-actin forward and reverse primers (10 μM), 2 μl of cDNA diluted 1:1 and 4 μl of nuclease-free water. Amplifications were performed with an initial step at 95°C for 1 min, followed by 40 cycles at 95°C for 15 s and 60°C for 40 s. A melting curve analysis of PCR products was performed from 60°C to 95°C in order to ensure gene specific amplification. UCP2 gene amplification was associated with β-actin as standard control. Changes in the UCP2 gene expression in the different samples were obtained in according to standard 2−ΔΔCt method [2]. UCP2 primer sequences: forward primer, 5’-AGCAGTTCTACACCAAGGGC-3’; reverse primer, 5’-AGAGGTCCCTTTCCAGAGGC-3.’ β-actin primer sequences: forward primer, 5’-ACCCGCCACCAGTTCGCCAT-3’; reverse primer, 5’-CGGCCCACGATGGAGGGGAA -3’. Specificity of UCP2 and B-actin primers was tested with conventional PCR that retrieved a single cDNA fragment of 230 bp and 128bp, respectively. The UCP2 cDNA fragment was also sequenced using automated methods on an ABI PRISM Genetic Analyzer (PE Biosystems). The results showed 100% homology with the Rattus norvegicus UCP2 gene. (TIF) [file pone.0265521.s003.tif]
